# Examining the Effects of Rabbit-Assisted Interventions in the Classroom Environment

**DOI:** 10.3390/ani10010026

**Published:** 2019-12-20

**Authors:** Marcell Molnár, Réka Iváncsik, Barbara DiBlasio, István Nagy

**Affiliations:** 1 Faculty of Agricultural and Environmental Sciences, Kaposvár University, Guba S. str. 40, 7400 Kaposvár, Hungary; molnar.marcell@ke.hu; 2 Faculty of Pedagogy, Kaposvár University, Guba S. str. 40, 7400 Kaposvár, Hungary; ivancsik.reka@ke.hu (R.I.); di_blasio.barbara@ke.hu (B.D.)

**Keywords:** rabbit-assisted intervention, level of anxiety, classroom, primary school

## Abstract

**Simple Summary:**

At the beginning of the first grade of elementary school, children generally show higher levels of anxiety, which continually decreases due to interactions with teachers. However, anxiety levels in children can also be decreased by using additional methods such as animal-assisted interventions in class. In this study, the efficiency of a rabbit-assisted intervention was examined in two first-grade classes with different backgrounds. In one of the classes, most of the pupils had special education needs. It could be concluded that rabbit-assisted interventions were particularly efficient where the anxiety levels of the children were high. This method seems to be useful in improving the efficacy of the educational ability of teachers.

**Abstract:**

The effect of rabbit-assisted interventions on the anxiety levels of first-grade children at a primary school was analyzed. At the beginning of our research, no rabbit-assisted intervention was applied for 6 weeks in order to establish the level of stress caused by the start of the education period. We then alternated 6-week-long periods with and without rabbit-assisted intervention. The level of anxiety in children was assessed every three weeks both in the assisted and non-assisted periods, using the State–Trait Anxiety Inventory for Children, STAI-C. One of the examined classes did not apply the integrating policy while the other class contained pupils with special education needs (called the integrating class). Rabbit-assisted interventions proved to be efficient, as anxiety level scores were significantly lower during the animal-assisted periods. The rabbits actively initiated encounters with humans, not only in the framework of animal-assisted interventions, but also at other occasions. In cases of discomfort, the rabbit retreated to the cage and stayed inside for a short time. The children displayed signs of pleasure when the rabbits approached them. The favorable effect of animal assistance was more apparent in the integrating class. It could be concluded that rabbit-assisted interventions were suitable for decreasing the anxiety levels of elementary school children, improving the efficacy of the educational ability of teachers.

## 1. Introduction

Animal-assisted interventions have been shown to have diverse results in several aspects. One of these aspects is the proven positive impact of animal-assisted pedagogy on the anxiety levels and the levels of depression of pupils. In most studies, animal assistance was based on dogs [1]. The presence of the animal, its spontaneous behavior, and its ability to socially interact promote educative as well as therapeutic processes [2,3,4]. 

Friedmann et al. [5] reported decreased blood pressure in children when they practiced reading out loud in the presence of a dog. In a similar study [6], anxiety in children related to reading out loud was more reduced by the presence of a dog compared to that of a friend or an adult person. 

AAP (Animal-Assisted Pedagogy) also known as AAE (Animal-Assisted Education) is a procedure in which a trained pedagogue, who is experienced in animal behavior and who is conscious of his/her own pedagogical purpose, conducts an educative process. Numerous studies in well-developed countries have been devoted to the investigation of classroom applications of Human–Animal Interactions (HAI) in the course of pedagogical practices. According to these, domestic and even non-domestic animals can be involved in the teaching process, both in a direct and an indirect way. Involving animals in the classroom has become particularly frequent in the educational and teaching programs for early childhood and for primary schools [7,8,9]. In reply to a series of on-line questionnaires collected in the USA in 2015, teachers listed several species that they have used in their educational programs, namely, fishes, guinea-pigs, hamsters, crabs, reptiles, rabbits, and even other unusual species such as ferrets [10,11]. 

The positive effects (e.g., the attitude of school children towards animal, the improved self-image of those pupils exposed to risks, and the improved attitudes towards school and adults) of involving animals in the classroom was also reported by Zassloff et al. [12] and Rud and Beck [13], and based on their results, the animals help the learning process as natural motivators. The spontaneous interactions between the animals and children result in “teachable moments”, increasing the efficiency of learning.

The use of the Animal-Assisted Interventions (AAI) for special pedagogical purposes (for special education of disabled children) has also been around for a long time, so it is no longer a peculiar technique in special education [14]. HAIs are well-established and possess elaborate protocols in the treatment of attention deficit/hyperactivity disorder (ADHD) or in the treatment of emotional and behavioral control of juveniles [15]. In Austria, it is already a legally-recognized option to involve the teacher’s own pet in AAIs in the classroom [16]. 

The stress-reducing effect of animals was mentioned by various authors who reported findings related to dogs [17] or involving animals in workplaces [18]. Animals interact with children, which provides comfort and can reduce stress and anxiety [19,20,21].

Among the different physiological parameters, animals affect heart rate and cortisol levels [22,23]. Barker and Dawson [24] justified by means of a self-reported test the effects of Animal-Assisted Therapy (AAT) on the anxiety ratings of hospitalized psychiatric patients.

According to Walter-Toews [25] among the 150 American and 74 Canadian societies performing AAT programs, the majority of these programs (94%) used cats or dogs. The proportion of rabbits, pocket pets (hamsters, gerbils, mice, guinea pigs) and birds was 28%, 15%, and 10%, respectively. 

There were similar proportions among the different species (dogs: 59.8%, cats: 22.5%, rabbits: 9.8%, and birds: 8.8%) reported by Schlote [26] and by De Santis et al. (dogs: 69.8%, horses: 32.3%, donkeys: 32%, rabbits: 16.2%, cats: 12.5%) [27]. There are some species (guinea pigs, chickens, ferrets, etc.) that can be used in AAI, but it has to be evaluated whether these species can also be used for AAE. 

In the present study, the animal species were chosen based on our previous study [28]. Our first choice was to use cats, which are easy-to-handle animals (house-trained), loved by children, and also able to solve different tasks. Unfortunately, cats’ behavior is not predictable, and cats can cause severe injuries to children. Cats also do not tolerate changing environments and their fur is an allergen. Human–cat interactions are sometimes conflicting [29]. Ferrets can also be house-trained animals with a friendly look, but they cannot be tamed without difficulties, and their bite can be dangerous [29]. Children also like dwarf hamsters, but they are crepuscular animals, and animal-assisted interventions would disturb their daily rhythm. Moreover, due to their small size, free movements cannot be allowed [30]. Tortoises can hold the attention of children only for a short period. The cannot learn tasks, and children do not stroke them. Besides, they are sensitive to the environment (e.g., temperature) [31]. Guinea pigs were used in several studies as AAI subjects as children like them [32,33,34,35,36,37]. Unfortunately, they are not house-trained. They can disturb the teaching with their noises and they are too small to move freely in the classroom.

In this study dwarf rabbits were chosen as the subjects of animal-assisted intervention because children like these animals, and they generally do no harm to children. They are quiet and do not disturb teaching. They can be house-trained, are capable of solving different tasks, and are not afraid of interactions with humans. We chose rabbits for our investigations since there is little related literature on them [38], and because rabbits are easily kept and cheap. Rabbits need no particular training as opposed to dogs, for example. Rabbits represent a much smaller challenge for the teachers than bigger animals. Also, children are keen to get involved with them, sometimes motivated by their former experiences with rabbits. 

Rabbits are generally popular among children because they can easily be socialized, their behavior is friendly, and their body gestures are unambiguous [39].

Some studies describing the effects of rabbits on humans are already published [40,41,42]. On the contrary, no publication is available examining the effect of AAI on rabbits. Based on our preliminary studies [43] rabbits showed large variability regarding suitability for AAI. Boldness test results showed that some individuals seek to establish human–animal interactions while other animals do not. This character seems to be heritable although it can also be modified by handling.

It is very important that rabbit-assisted interventions would not cause any damage to the animals. This objective can be achieved by performing selection on tameness and by handling. Handling is a method that encourages the rabbit to tolerate human contact and reduces periodical stress. The plasticity of cognition of environmental effects is more pronounced during some phases of development [44], called socializing periods. Contact with humans during these periods hinders the development of fear against humans during latter life periods [45].

According to several studies related to pigs, sheep, and cattle [46,47,48], different treatments may influence the behavior of the domesticated animals and animal–human interactions (from the aspect of fear and approachability). In rabbits, touching the animal by hand, according to Hudson et al. [49], decreases fear if carried out during the first week after birth.

Pongrácz and Altbacker [50] reported that the repeated treatments (especially during the first week after birth) positively affected the welfare and behavior of the caged rabbits, and the kits show less fear against humans if the treatments occur shortly after or before nursing. Treatments at early life stages substantially influenced reactivity of rabbits in behavior tests [51,52].

The rabbits treated in different periods were evaluated by means of an open field test [53]. The inter-litter variance was high and the findings suggested that selective breeding may be a superior method compared to handling at a young age if the objective is to have rabbits without fear for humans. This was also justified by Daniewski and Jezierski [54].

## 2. Materials and Methods

### 2.1. Animals and Experimental Design 

Aiming to evaluate the efficiency of animal-assisted education, our research was performed with the participation of first-grade primary school children from two different classes. The main objective of the study was to decrease stress in children related to the start of the elementary school. We chose the two classes based on the frequency of children with special education needs (SEN) and learning and behavioral disorders (BTM). The so-called integrating schools are characterized by the large proportion of BTM and SEN children originating from poor and poorly educated families. Children with these kinds of problems have difficulties in coping with the school environment. There is no integrating program as such, but these children have well-adjusted classmates who help them develop. The other school has very few BTM or SEN children. These latter children are from wealthy and well-educated families. In the classes of the integrating school, the frequency of the SEN and BTM children is around 70% while in the other school it is less than 1%. The teachers accepted the additional tasks related to the rabbit-assisted interventions. In the different years there was only one class so it was not possible to use a control class (with special training but no animal assisted intervention).

The numbers of school children in the evaluated classes were 22 (in the integrating school) and 29 (in the majority school) but only the results from those children who participated in all special trainings were used (one training per week when rabbits were present in classes and altogether 12 trainings) thus the records of 8 (in the integrating school) and 19 (in the other school) children were evaluated.

Six-week-long periods with and without rabbits in the classes were alternated in order to determine the magnitude of the effects of the rabbits’ presence.

At the beginning of our research, no rabbit-assisted intervention was applied for 6 weeks in order to establish the level of stress caused by the start of the education period. Then, six-week-long periods with and without rabbit-assisted intervention were alternated. These periods did not disturb any school program or vacation.

Before starting the animal assisted developmental program, the school children were evaluated by special education teachers and psychologists. The adaptive, motor, language, cognitive, and counting skills development was measured and used for creating the capability profile. The objective was to improve the skills that were less developed. In the course of the analysis, general school readiness (Difer) and WISC (IV) tests were performed. The diagnostic progress test (Difer) is a system of tests evaluating the critical elementary skills at elementary school age. The so called development indicator monitors the critical elementary skills between the ages of 4 and 8 years. By applying these systems, criteria-oriented skill development is possible. Difer is a compulsory element of public education used in the system of measurement and evaluation. At the beginning of the first year, the elementary schools measure the children and identify those pupils where detailed evaluation by means of Difer could be justified. WISC administration requires 45–65 min. It generates full scale IQ, characterizing the intellectual capabilities of school children. It includes five different index parameters: Verbal Comprehension Index, Visual Spatial Index, Fluid Reasoning Index, Working Memory Index, and Processing Speed Index. These indices represent the capabilities of children at discrete cognitive areas. This technique can only be used by psychologists.

University students of special education held a 45-min-long cognitive training, each topic was focused on one kind of animal (rabbits). Afterwards, the children could experience direct physical contact (e.g., stroking) with the animals. The topic of the classes was in accordance with class schedules.

During the first meeting, the rules and regulations connected to animal welfare and human behavior towards rabbits were explained. Animal-assisted cognitive trainings were accomplished in groups using various methods. The areas that were falling behind (motor, visual, auditory, social communication skills, tactile sensation) were improved by means of exercises.

The rabbits could freely approach any children and the chosen child could also stroke the rabbit. Those children who gave correct answers to the exercises could approach and stroke the rabbit. The rabbits could freely move and could also approach those children who did not give the correct answer. However, the possibility of stroking the rabbit more times was inspiring for the children. The most important element of the development was the direct physical contact with the animals. The rabbits could freely approach any children and the chosen child could also stroke the rabbit. After the cognitive training program was finished, the development of the children was re-evaluated.

The visual contact between the children and the rabbits was continuous, and in addition, once a week a special course was organized for the pupils: The level of anxiety in children was assessed every three weeks both in the assisted and non-assisted periods, using the State–Trait Anxiety Inventory for Children, STAI-C [55], a widely used report form identifying problem behavior in children. Standard scores were elaborated based on the data of Hungarian children [56]. Thus, the obtained scores are not utilized for diagnosing diseases but to establish the difference compared to the mean. Answering the 20 questions related to anxiety with the responses “never”, “sometimes”, and “frequently” correspond to scores of 1, 2, and 3, respectively. The possible maximum score of the test is 60. Pupils with scores over 35 are classified “anxious/stressed children”, between 30 and 35 scores “slightly stressed children”; below 30 “of normal anxiety/stress level”. Only the results of children (altogether 27) participating in all tests were considered.

During the animal-assisted interventions, one rabbit was present in each class, so altogether, four (lion-headed dwarf) rabbits were used in this study. The rabbits came from a line that had been selected for calmness and stress tolerance for generations. Besides, these rabbits were handled shortly after their birth in order to improve the latter contact to humans. All rabbits were females (0.5–1-year old) in order to avoid the territorial marking (urine) of the male rabbits in classes.

The rabbit cage was placed on a platform (its height was 10 cm) beside the teacher’s desk because all children could continuously see the rabbit. This place is the calmest area of the class because movements of the children are less likely to disturb the rabbit. During the day, the rabbit had several occasions to leave to cage after the cage door was opened by the teacher.

The cage (Dimension: 95 × 57 × 46 cm) has a deep colored plastic bottom on which a metal mesh was placed. The mesh was coated with special corrosion-resistant paint, and could be completely lifted from the front side, for easier access. This also made daily cleaning easier. The cage for rabbits was roomy inside and came with all the necessary accessories for the rabbit, including a hay container, water nozzle, food bowl, little house for sleeping, and rabbit toilet (filled with wood pellets) all made of plastic.

The rabbits received a complete diet and the food was made in pellet form, so that selective eating was prevented. The pellets were rich in fibers for good intestinal functioning. In addition, every day the rabbits received hay and fresh vegetables. The rabbits were fed two times every day (in the morning and at the end of the teaching period) by volunteer children who also cleaned the cage and changed the spoiled litter. Water was available ad libitum from nipple drinkers and hay was also continuously available from hay racks. Environment enrichment was accomplished with the use of gnawing sticks and the cages were also equipped with mineral supplementary blocks.

Prior to animal-assisted interventions, all rabbits were checked by veterinarians (blood, feces), and the rabbits were vaccinated against Myxomatosis and Rabbit Haemorrhagic Disease (RHD) and also treated against parasites. The claws were shortened to avoid injuries. Feces was continuously monitored to identify possible health problems. Potential health problems could be caused by Salmonella and Campylobacter infections, allergen reactions, and worms [25]. All rabbits were free from zoonosis and parasites.

According to the daily routine of the rabbits, they stayed calmly in the cage and consumed food and water. When their cage was placed on the playing carpet, they left the cage, moved around the class, and received strokes from the children. After varying amounts of time, when they got tired, they moved back to the cage, which was always respected by the children.

Generally, the rabbits could freely move in-and-out of the cage during the teaching period (07:30–17:30) except in some cases (e.g., during game time ) when, for the sake of the rabbit’s safety, staying in the cage was necessary. Based on the results of preliminary analysis, the stress levels (measured by cortisol levels) of the rabbits was higher during transportation compared to that of staying in the cage. Based on this finding, it was decided that leaving the rabbits in the cage was preferable compared to everyday transportation. Nevertheless, for the weekends, the rabbits were transported to their stock and were not left in the classroom without any surveillance. Thus, during the weekends, the rabbits could interact with their conspecifics and could go outside. The analysis of the rabbits’ behavior was based on the publication of the Royal Society for the Prevention of Cruelty to Animals (RSPCA) defining the body language of rabbits [57] and by the parameters reported by Mayer [58]. According to Mayer, the signals of stress were as follows: modification of behavior or physiology, inexplicable aggression, cage biting, increased or decreased feed consumption, strange movements (e.g., circling), fear, or depression. The behavior of the rabbits was monitored but no signals of stress were found. No video recordings were made to analyze the behavior of rabbits because that would disturb the teaching. The rabbits could freely choose between staying inside or outside of the cage in the classroom. They generally stayed outside, and only entered the cage for food consumption, defecation, or resting. They spent a lot of time outside the cage, freely moved around in the classroom, and also rested under the teacher’s desk.

During the cognitive trainings, the rabbits had to tolerate strokes from the children, but as they originated from a selected strain this did not cause any problem. In the course of selection (which lasted for four generations) the rabbits, suitable for the animal-assisted interventions in classes, were chosen based on a boldness test [59,60] and by measuring cortisol levels. The animals actively initiated encounters with humans, not only in the framework of animal-assisted interventions but also at other occasions. In cases of sudden noises, the animal retreated to the cage and stayed inside. The children received the approach of the rabbits with pleasure.

### 2.2. Statistical Analysis

The effects of the different periods (assisted vs. non-assisted), sex of the children (male vs. female), and the school system (integrating vs. non-integrating) on the anxiety scores were analyzed using Generalized Linear Model GLM analysis taking into account that the same children were evaluated repeatedly. Statistical analysis was performed using SAS 9.4 software using the PROC MIXED procedure.

## 3. Results 

Results of the GLM analysis are given in Table 1. The procedure calculated the corrected means for the different factor levels and estimated the differences among them. 

Based on the results, all the examined factors significantly affected the anxiety score. The largest effect was found based on the type of school, where the pupils of the majority school showed substantially lower anxiety scores than those children in the integrating school. In general, male pupils had lower anxiety than females. Among the analyzed factors, the presence of the rabbits in the class had the smallest effect on anxiety, but the presence of the rabbits significantly decreased the anxiety score by around 8%, on average.

## 4. Discussion

Based on the findings of this study it can be stated that the presence of the rabbits significantly reduced the stress (related to the starting of the elementary school) of children. Similar results were reported by Havener et al. [61] based on 7 to 11-year-old children where the stress related to dental treatments could also be reduced with animals.

The level of anxiety was reduced during the periods of cognitive trainings. The magnitude of the animal-related effect on the anxiety was different in the analyzed schools, but it was not surprising as anxiety levels of children of the two schools were different from the onset of the study.

In the integrating school (with SEN children) the decrease of the stress was larger after the third cognitive training compared to the other school. However, it must be noted that animal-assisted interventions can be effective if the initial anxiety level is high. The cognitive trainings applied in this study were suitable to decrease the anxiety level in schools, helping the teachers’ work become more effective.

Concerning the effect of the animal assistance, some latency can be observed. Examining the decrease of anxiety caused by small animals, O’Haire et al. [35] analyzed typically-developing children diagnosed with Autism Spectrum Disorder (ASD) and reported that ASD children showed higher activity and stress in every situation except when small animals (guinea pigs) were present.

The animal-assisted interventions have a positive influence on motivation, self-efficiency, attention, self-control, and group-related competences [10]. However, it is important to keep in mind that tradeoffs between humans’ and animals’ health should be avoided, and synergistic benefits should be achieved on both sides during animal-assisted interventions [62]. According to Menna et al., parameters that need to be further investigated include interspecific relationships and the factors influencing them, as well as the inter-specific relational competences of the species and the individual animal chosen and its relationship with the handler [63].

**Figure 1:**
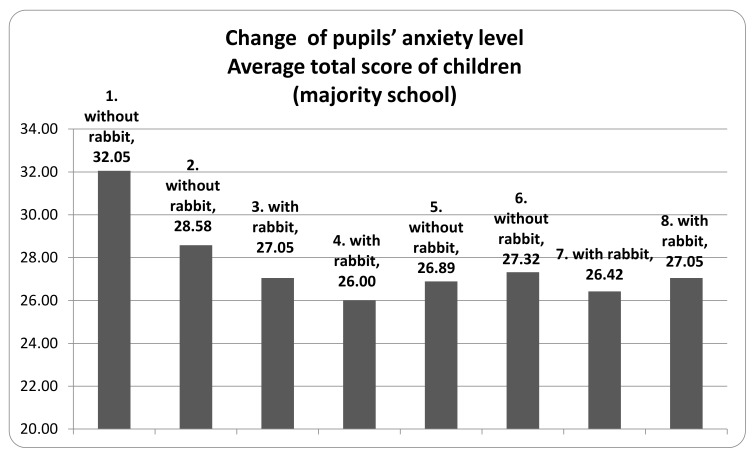
Change of pupils’ anxiety level.

**Figure 2:**
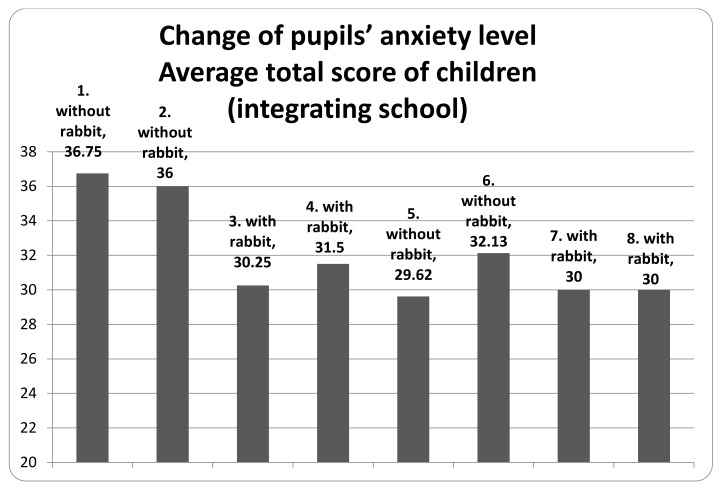
Change of pupils’ anxiety level.

At the beginning of the teaching period, the level of anxiety in one of the examined classes remained under 35. Nevertheless, there was an apparent decrease in the scores of the first and second evaluation (Figure 1), which was the result of the activity of the teachers who helped the children to adapt to elementary school education. Afterwards, a slight fluctuating pattern could be observed where the scores were somewhat higher in the non-assisted compared to the assisted periods. In the other class (which belongs to an integrated school), the anxiety score tendencies were different. The teachers’ activities to help with adaptation at the initial period were less effective. However, the fluctuating pattern of the scores in the assisted and non-assisted periods was more pronounced than in the other class with no integration policy. Despite of the favorable effect of the rabbit-assisted intervention, the level of anxiety of children originating from the integrating school (Figure 2) was constantly higher (“anxious/stressed” to “slightly stressed”) than in the other class (“slightly stressed” to “normal”) with no integration.

**Figure 3:**
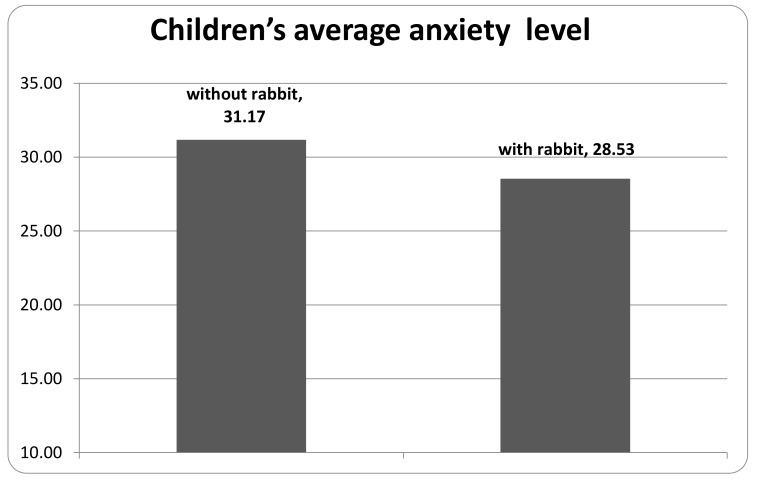
Children’s average anxiety level in the rabbit-assisted vs. the non-assisted periods.

Pooling the scores of all the animal-assisted and control periods (Figure 3), the score of the assisted period was lower by 2.64, which corresponds to 8.45%. The meaning of this finding is that on average the anxiety level could be decreased from “slightly stressed” to “normal”. 

**Figure 4:**
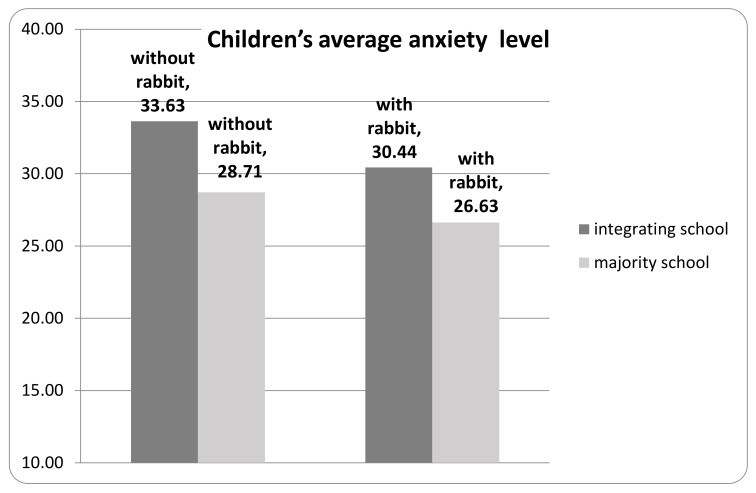
Children’s average anxiety level in the majority and the integrating schools.

The effect of the assisted periods was larger in integrating schools (9.48%) than in the non-integrating schools (7.24%) (Figure 4).

School children showed not only highly variable initial scores, but also the magnitude of score changes was variable following the assisted and non-assisted periods. Based on their initial anxiety scores, pupils were classified into three groups. Then, the score changes of these groups were examined. In the “stressed” group (Table 2) the decrease of the anxiety scores was 5.06% during the assisted periods (36.19) versus the non-assisted periods (31.13). Consequently, classification changed from “stressed” to “slightly stressed”. Some of the children decreased their anxiety level scores by 19–20 by the end of the intervention, and the average score differences between the assisted and non-assisted periods was 10.

Lower improvement was found among the “slightly stressed” children (Table 3), where the anxiety scores were 1.94% lower in the assisted periods. However, some children showed 7% improvement, while others did not show any change in the anxiety level scores.

Among the normal category students (Table 4), minimal (0.68%) changes could be observed. For most of these children, no change in stress level was registered. Moreover, in some cases, a slight negative effect of the presence of the animals was registered. For this group, the animal-assisted training seems to be unnecessary. Occasionally, the presence of the rabbit may even disturb them in their learning activity. 

## 5. Conclusions

Based on our pilot research, the effect of rabbit-assisted activity was beneficial on the anxiety of first-grade pupils. The favorable effect of animal assistance was dependent on the level of anxiety at the initiation of the research. Most pronounced improvements were observed for the “stressed” children, while the improvements were lower and negligible, respectively, for “slightly stressed” and “not stressed” pupils. The animal assistance was more effective in integrating schools. It can be concluded that rabbit-assisted interventions were suitable for decreasing the anxiety levels of elementary school children, improving the efficacy of the education activity of teachers. There are ongoing investigations in three schools (five classes). The rabbits are evaluated by means of boldness tests, children by saliva tests, and the rabbits’ feces samples are taken weekly (before and after animal assisted interventions) in order to determine the level of stress. Hence, the effect of the AAI on the anxiety levels of children and the level of stress of the rabbits can be evaluated simultaneously. 

## Figures and Tables

**Table 1:** Results of the GLM analysis

Factor	Estimated Differences Among the Factor Levels	Significance of the Differences
School (Majority-Integrating)	−7.43	<0.0001
Rabbits (Present-Not present)	−2.41	<0.0001
Sex (Male-Female)	−5.69	<0.0001

**Table 2:** Group of anxious children.

Period	1st Child	2nd Child	3rd Child	4th Child	5th Child	6th Child	7th Child	8th Child	
Ø	48	40	50	44	52	37	39	41	
Ø	46	40	50	43	41	26	34	30	
R	34	31	32	40	40	31	31	27	
R	42	26	37	38	37	21	23	25	
Ø	36	21	40	40	36	20	22	24	
Ø	41	22	41	39	36	27	25	27	
R	46	20	35	31	36	21	21	29	
R	41	20	37	40	39	20	20	25	
									avg
Ø avg	42.75	30.75	45.25	41.50	41.25	27.50	30.00	30.50	36.19
R avg	40.75	24.25	35.25	37.25	38.00	23.25	23.75	26.50	31.13
d (%)	2.00	6.50	10.00	4.25	3.25	4.25	6.25	4.00	5.06

Ø—rabbitless period, R—rabbit-assisted period, Ø avg—average of rabbitless period, R avg—average of rabbit-assisted period, d (%)—difference in %.

**Table 3:** Group of less anxious children.

Period	9th Child	10th Child	11th Child	12th Child	13th Child	14th Child	15th Child	16th Child	
Ø	34	32	32	31	31	33	33	34	
Ø	36	29	21	28	29	29	31	25	
R	33	25	24	26	28	29	27	22	
R	29	38	21	20	28	32	28	28	
Ø	32	31	26	20	22	32	27	29	
Ø	36	32	29	20	26	36	30	23	
R	24	31	28	20	28	35	28	27	
R	24	31	27	20	31	35	26	24	
Ø avg	34.50	31.00	27.00	24.75	27.00	32.50	30.25	27.75	29.34
R avg	27.50	31.25	25.00	21.50	28.75	32.75	27.25	25.25	27.41
d (%)	7.00	−0.25	2.00	3.25	−1.75	−0.25	3.00	2.50	1.94

Ø—rabbitless period, R—rabbit-assisted period, Ø avg—average of rabbitless period, R avg—average of rabbit-assisted period, d (%)—difference in %.

**Table 4:** Group of normal stress level children.

Period	17th Child	18th Child	19th Child	20th Child	21th Child	22th Child	23th Child	24th Child	25th Child	26th Child	27th Child	
Ø	24	29	28	24	29	29	22	24	29	28	26	
Ø	22	39	20	21	28	29	22	28	29	29	26	
R	20	36	22	22	30	28	20	25	26	20	27	
R	26	33	20	21	29	33	20	22	26	20	23	
Ø	21	36	22	22	32	25	22	36	27	20	27	
Ø	25	33	20	20	31	26	23	33	26	22	27	
R	25	38	20	20	32	32	21	22	29	20	23	
R	25	42	20	20	35	33	21	26	28	20	24	
Ø avg	23.00	34.25	22.50	21.75	30.00	27.25	22.25	30.25	27.75	24.75	26.50	26.39
R avg	24.00	37.25	20.50	20.75	31.50	31.50	20.50	23.75	27.25	20.00	24.25	25.57
d (%)	−1.00	−3.00	2.00	1.00	−1.50	−4.25	1.75	6.50	0.50	4.75	0.68	0.68

Ø—rabbitless period, R—rabbit-assisted period, Ø avg—average of rabbitless period, R avg—average of rabbit-assisted period, d (%)—difference in %.
